# Army Veterinary Corps

**Published:** 1900-06

**Authors:** 


### ARMY VETERINARY CORPS.

DISCUSSION IN THE UNITED STATES SENATE ON THE ADOPTION OF SENATOR KENNEY'S AMENDMENT TO THE BILL FOR THE EFFICIENCY OF THE ARMY, WHICH BECAME SECTION 14 OF SAID BILL, S. 4300 (COMMONLY KNOWN AS THE ARMY REORGANIZATION BILL).

[Extracts from the *Congressional Record*, May 4, 1900.]

Mr. Kenney: I offer the amendment which I send to the desk to come in on page 7, line 18, after the word "transfer."

The President pro tempore: The amendment submitted by the Senator from Delaware will be stated.

The Secretary: On page 7, in line 18, after the word "transfer," it is proposed to insert:

That the Veterinary Corps shall consist of—

A chief veterinarian, with the rank, pay, and allowances of a colonel, United States Army.

An assistant chief veterinarian, with the rank, pay, and allowances of a major, United States Army, to be promoted in 1905, after competitive satisfactory examination, from the grade of veterinarian and captain.

Four veterinarians, with the rank, pay, and allowances of a captain of cavalry, to be promoted in 1903, after competitive satisfactory examination, from the grade of assistant veterinarian and first lieutenant.

Ten assistant veterinarians, with the rank, pay, and allowances of a first lieutenant of cavalry, to be promoted, after satisfactory examination, from the grade of assistant veterinarian and second lieutenant after one year's service in this grade.

Twenty assistant veterinarians, with the rank, pay, and allowances of a second lieutenant of cavalry, to be appointed after satisfactory examination: *Provided*, That these twenty positions shall include the veterinarians, first class, provided for in the act of March 2, 1899, who have passed satisfactory examinations, and also the six veterinarians, second class, who are now employed in the army under said act of March 2, 1899.

All rules and regulations governing the Veterinary Corps shall be made by the Secretary of War, and the chief veterinarian shall report directly to that officer.

For pay of officers of the Veterinary Corps, $33,500.

Mr. Kenney: Mr. President, I shall not detain the Senate at any length by remarks on the amendment which I have just offered.

In any scheme for the reorganization of the Army of the United States there is no question that deserves more consideration than does that of a properly organized and commissioned veterinary corps. This country is alone in all the world in maintaining a great army without a properly organized and commissioned veterinary corps. In our Army to-day veterinarians are nothing more than civilian employés, hired from day to day, without any responsibility, and practically without authority.

The amendment which has just been read would increase the expense necessary to that corps over what is to-day being expended for the civilian service not more than $6000, while it would give to the service a corps commissioned and with all the responsibilities and with all the powers that go to commissioned officers.

I fail to understand what legitimate objection can be urged to the substitution in the Army of the United States of a corps like the one proposed by the amendment just read for that which to-day obtains in our service.

I have made a statement of the strength of the armies of the leading countries of the world, together with the number of veterinarians employed in those armies, which are as follows :

STAFFS OF VARIOUS ARMIES, JANUARY, 1899.

| Country. | Strength active army. | Medical officers. | Strength cavalry. | Veterinary officers. |
|---|---|---|---|---|
| Austro-Hungary | 305,735 | 1385 | 47,380 | 180 |
| Belgium | 51,302 | 150 | 6,064 | 35 |
| Denmark | 13,734 | 55 | 1,390 | 14 |
| France | 617,959 | 1485 | 80,048 | *a*432 |
| Great Britain | 222,301 | 1580 | 17,586 | 136 |
| Greece | 24,076 | *a* | 4,877 | *a* |
| Holland | 78,093 | 184 | 4,193 | *b* |
| Italy | 230,760 | 691 | 23,507 | 189 |
| Japan | 87,560 | *b* | 3,500 | *b* |
| Prussia | 443,516 | *c* | 53,270 | *d* |
| Roumania | 127,984 | 73 | 16,053 | 44 |
| Russia | 868,672 | *c* | 90,000 | *c* |
| Spain | 128,153 | 793 | 14,376 | 236 |
| Sweden | 38,854 | *c* | 5,269 | *c* |
| Switzerland | 144,822 | *c* | 3,972 | *c* |
| Turkey | 286,000 | *c* | 21,800 | *c* |
| United States | 100,000 | *f*196 | ..... | *e* |

*a* Increased in 1899 to 603.—*Thierartze-Wochenschrift.*

*b* Number not given.

*c* Classification given by corps, division, brigade, and regimental staffs ; to each of which medical officers and veterinarians are attached.

*d* Increased in 1899 to 501.—*Thierartze-Wochenschrift.*

*e* Irregular number of civilian employés.

*f* Some 450 additional acting assistant or contract surgeons are also employed.

And so, without reading this list, Mr. President, I may say every civilized country in the world does maintain, organized and commissioned, a proper veterinary corps.

Mr. President, in support of my amendment, I desire to have incorporated as a part of my remarks letters from distinguished officers of our Army, beginning with General Merritt. I will not read the letters now. They are from General John R. Brooke, General James H. Wilson, General J. C. Breckenridge, General Wallace F. Randolph, colonel of the First Artillery; General Charles King, formerly captain of the Fifth Cavalry, now brigadier-general, United States Volunteers; Colonel O. L. Hein, lieutenant-colonel of cavalry and commandant of cadets; General James A. Beaver, ex-Governor of Pennsylvania; J. G. C. Lee, colonel and assistant quartermaster-general, United States Army; Colonel D. D. Wheeler, deputy quartermaster-general, and Major John W. Pullman, quartermaster, United States Army. All of these officers have not only approved this by word, but have written strong[1] letters favoring the establishment of such a corps.

An amendment offered by me to the Army Appropriation Bill and also one offered to the bill introduced by the distinguished Senator from Connecticut [Mr. Hawley] went to the Committee on Military Affairs, but had no consideration, as I understand. I now propose this amendment to this bill, which, I understand, is for the reorganization of the Army and for the betterment of the service. If its title means anything, then there is no branch or arm of the service that needs more consideration than does the Veterinary Corps.

I hope that Senators in passing upon this question will take into consideration that in the establishment of this corps it is not going to cost the Government of the United States a sum larger by more than $6000 than that which is to-day being paid for the kind of service the Government is receiving.

In this connection I might say that there is not to-day in the Army service veterinarians who are able to give that consideration and examination to the great question of Army foods that is required. Many will remember the very disagreeable criticism that was passed upon the administration of our Army during the Spanish-American war in regard to the food that was furnished to our armies. I desire to say—and I believe it to be true—that if we then had had a commissioned veterinary corps under proper regulations, that criticism would never have been had.

It is fresh in the minds of many Senators what great loss the Government sustained in the death of the cavalry and artillery horses stationed in Florida. That loss was largely due to the lack

[1] The letters referred to have already been furnished to all veterinarians in pamphlet form.—Ed.

of veterinary treatment. In the United States to-day the value of army animals is so large that it seems to me any legislation which would aid in the preservation and protection of the property of the United States in these animals would be the strictest sort of economy.

I do not desire to go into a more extended argument in favor of this proposition.

Mr. Stewart: Mr. President, I believe this is about as important a matter as any other connected with the Army. Having some knowledge of veterinarians in general, I think that that service ought to be improved. The men who are hired as veterinarians do not generally have such an education as would fit them to act in the examination and purchase of horses for the Government and in the care of such horses. The Government suffers great loss, and the Army is crippled and inefficient. On the mere matter of purchasing five or six or eight or ten thousand horses the Government will save more than the amount it would pay for a proper organization in the way of a veterinary corps to examine the horses.

Mr. Proctor: Will the Senator from Nevada allow me?

Mr. Stewart: Certainly.

Mr. Proctor: I think the veterinarians are now examined. Their pay has been increased, so that the veterinarians of the first class receive $125 a month, the pay of an officer. Those of the second class receive $75 a month. I know it has been insisted upon for a long time that they should be graduates of veterinary colleges, and they are examined, I am sure, before they are taken into the service.

Mr. Stewart: It may be sufficiently provided for already, but my observation is to the contrary. I have heard a good deal of complaint in regard to it. I know that during the civil war the Government was largely imposed upon by the want of an experienced, organized veterinary force. If it is organized, then in order to make it efficient there should be an inducement to men of the first class to educate themselves, because in that regard a high standard of service is important to the efficiency of the Army. If it is thoroughly provided for, I have nothing further to say, although I know it is of great importance.

Mr. Proctor: I will say to the Senator that there has been an entire change since the civil war. Then they had about the pay of a sergeant. Now those of the first class have the pay of a commissioned officer, a first lieutenant.[1]

[1] Error. A Second Lieutenant.—Ed.

Mr. Kenney: I desire to state that I understand there are but five or six veterinarians in the United States Army to-day who have passed an examination, and I think about three or four, not exceeding five, who are in the service by reason of long service, who failed to pass an examination.

Mr. Foraker: I desire to ask the Senator having this bill in charge what is the distinction between the classes? The bill speaks of veterinarians of the first class and veterinarians of the second class.

Mr. Proctor: Those of the first class are those who have been longer in the service and are better entitled for that reason and for their proficiency to have that higher position.[1] Of course, that is open to the promotion of those of the second class.

Mr. Foraker: It does not imply a difference, then, in the qualifications, I presume.

Mr. Proctor: Not necessarily.

Mr. Foraker: All are supposed to be educated and capable and to have passed the examination, I presume.

Mr. Kenney: I will ask the Senator in charge of the bill one question. That is whether to-day there is or not a commissioned veterinarian in the United States Army?

Mr. Proctor: They are not commissioned officers. Those of the first class are paid the pay of a commissioned officer.

Mr. Stewart: Has this matter been considered by the Quartermaster's Department, which has particular connection with it? Has it been considered by the General of the Army or those having the matter particularly in charge? Have they been consulted in regard to this matter?

Mr. Proctor: This proposed amendment was before the Military Affairs Committee in the form of a bill, and was carefully considered, and I for one, and I think other members of the committee, visited the War Department and asked whether it was a thing desired for the Army, and they insisted that the present arrangement was entirely satisfactory.

Mr. Cockrell obtained the floor.

Mr. Gallinger: I will ask the Senator who insisted that that was the fact, inasmuch as almost all the higher officers of the Army have written letters and personally have said that they desire this to be accomplished?

Mr. Kenney rose.

[1] Error. The second class are those who failed in examination.—Ed.

Mr. Proctor: The Senator from Delaware, I am sure, has those letters in his possession.

Mr. Kenney: Yes, sir.

The President pro tempore: The Senator from Missouri was recognized.

Mr. Cockrell: Mr. President, the Committee on Military Affairs considered the general bill creating this corps of veterinary surgeons and voted against recommending its passage. I am opposed to the amendment, and I wish to say to the Senate that they probably have very little conception of the pressure that is brought to bear upon the Committee on Military Affairs to create additional corps in the Army. There are proposed a veterinary corps with a colonel at the head of it, a dental corps with a colonel at the head of it, a nurse corps with a colonel at the head of it.[1] All these have been attempted to be ingrafted onto the Army to create separate and independent corps, and the Committee on Military Affairs thus far has not deemed it necessary to make any of those additions.

We have not recommended a veterinary corps because we do not believe a corps organization for the veterinary service is necessary. We already have under existing law veterinarians, two for each regiment of cavalry, and have had ever since the 3d of March, 1899. They are provided for in the bill that was passed for the reorganization of the Army. One of them has the pay of a lieutenant and the other one has $75 per month. In this bill we create veterinarians for the horse artillery. I suppose there is no necessity that the heavy artillery should have veterinarians when they do not use horses.

There are two veterinarians of the first class and two of the second class, the first class receiving the pay of a lieutenant, the second class $75, and they shall have the pay and allowances of veterinarians of cavalry of corresponding classes. We think this is an ample provision for the veterinary service. As I understand it, these veterinarians to be appointed have to be examined by a board to be constituted by the Secretary of War. I know a number of veterinarians from Missouri who were seeking these places, and on presenting the applications to the War Department they said they would make no appointments—that was last spring—until they had adopted rules and regulations and a board for their examination. I have not asked whether that has been done

[1] These latter statements are incorrect.—Ed.

or not, but I presume it is in operation to-day, and that every veterinarian who has been appointed since the passage of the act of 1899, or who will be appointed under the provisions of this bill, will be those who have passed a satisfactory examination. I doubt not that examination will require that they shall be graduates of a veterinary institution of this country or Canada.

Mr. President, we can see no necessity for a colonel to have charge of veterinarians throughout the Army. Those two veterinarians to each regiment go with the regiment. They are there; the horses are there; they are there to attend to them; and in the artillery the veterinarians will be in the same way. We cannot see any necessity for or any benefit or any advantage from this. The only thing, it seems to me, will be the creation of a few officers with rank and pay and the privilege of retirement, and I do not believe that is either fair or just. It is purely a civilian employment.

Mr. Wolcott: Mr. President, I do not think the advocates of this amendment should be driven from its support because of the suggestion of the Senator from Missouri and others that opening the door to the appointment of a veterinary staff would likewise lead to the appointment of a dental staff. It would not frighten me, for I recognize in the dental profession a body of educated and cultivated gentlemen whom I should be very glad to have represented with official rank in the armies of our country, especially since we are reaching out to distant colonies where treatment such as is obtained at home is impossible. But in the consideration of this amendment we need not dwell upon the possibilities of other open doors.

Mr. President, the fighting of this world, as is now being demonstrated in South Africa, is largely cavalry fighting. I think that it is becoming more and more evident to every student of wars, and if it is, there is no profession in the world that needs such encouragement and such advancement as that of the veterinarian, who can see that the horses of the Army are fit to fight with.

There is not another civilized country in the world that does not recognize veterinarians and give them commissions. It is true we have a few veterinarians. They are called farriers, I think, in the Army, and we put them down in the rank of privates or sergeants. We pay one of them, it is true, the salary of a first lieutenant, and we give the other $75 a month, but that does not compensate a man who is a member of an honorable and educated and an intelligent profession. As was said here this morning by my colleague in reference to another subject, it is the rank that counts. I think that a little consideration will convince every member of the Senate, who is not already convinced, of the great importance of the service which the professional veterinarian renders and might still further render in the armies of the United States.

It is not alone in the treatment of the livestock of the Army. He deals also with the food that goes to our troops. He passes the meat on inspection. Every pound of fresh meat that is served to our soldiers passes under his intelligent inspection. All cattle that are bought upon the hoof and carried with the Army, to be killed as the necessity requires, must be treated by him; and any man who knows of the danger of contracting disease and sickness from diseased cattle can realize somewhat of the necessity that rests upon the Government to see that intelligent men deal with this question.

This Government has now, in times of peace, so far as its use of cavalry is concerned, more than $3,000,000 invested in horses and mules. Unintelligent shoeing, unintelligent acclimatization, unintelligent treatment, ignorance in the purchase of horses, not only costs us hundreds and may cost hundreds of thousands of dollars a year, but in time of war would lead to endless and disastrous consequences. In England the head of the service ranks as colonel. In Germany they enter as subordinate officers and are promoted as high as the rank of major. In France and Italy and every other country the veterinarian is recognized by his official title.

I tell you, Mr. President, this whole question is not, in the minds of those who are considering it, I fear, a question, as to the necessity of the service and the necessity of the intelligence of it, but it is a question whether or not the man who is vulgarly called a horse doctor ought to associate with officers who may be graduates of West Point.

Mr. President, we do not realize the growth that has taken place in that profession. There are to-day veterinary surgeons graduates of every college and university in the United States. I know men graduates of Harvard University living in Massachusetts who are to-day devoting their lives to the veterinary service. If it were not for the presence here of distinguished alumni of Harvard University I would almost be inclined to say that the flower of the university largely went into the veterinary service. I know graduates of Princeton and other colleges who adorn the profession.

All these professions are matters of growth and slow growth.

To-day the medical profession is recognized as one of the noblest and best and most sacrificing methods by which a man may devote his life to the cause of his country. It is not a hundred years since skilled physicians used to prescribe powdered snail shells for disease and certain herbs plucked at a certain stage of the moon's phases. There are to-day men travelling around in some of the Southern and Western States, physicians admitted to the profession, with madstones in their pockets to cure hydrophobia. The profession is a noble profession, and it is growing, and it is recognized, and we are proud to have our brothers and our relatives and our friends members of it.

So the veterinary profession is growing, and if it be noble to devote one's self to ameliorating human suffering, it is greater and nobler in some respects to devote your life to the amelioration and the relief of the sufferings of dumb animals. That is what is going on in the profession to-day. Men from colleges and universities come out from them intending to devote their lives to the welfare of mankind and the advancement of the world. They find their mission in looking after the dumb animals who cannot tell their sufferings, and in trying to make them better able to bear the burdens which man puts upon them.

I say, Mr. President, that it is not fair to relegate these men as farriers and put them in as non-commissioned servants. An American officer is a gentleman by his conduct and not by his designation. The skilled veterinarians of the Army are as much entitled to the rank of officers as West Point men are, if they are gentlemen and fill the duties they are called upon to perform. The officers of the Army do not object to associating with them. From General Sheridan's time to now there has been an earnest effort by every general who has ever fought with cavalry to have the veterinarians recognized as an official branch of the service. I am surprised to hear the Senator from Vermont say that he made a call on the War Department and found somebody in charge there opposed to it. For one, I should be glad to know who it is at the War Department who does not think a veterinarian ought to have the official rank and pay and be entitled to associate officially with other officers of the Army.

So I say all of us recognizing the usefulness of this profession, all of us recalling the fact that the officers of the Army do not object to the veterinarians having name and rank and place as officers of the Army, we can afford to give them the title and the rank, and it means no increase of appropriation.

Mr. Proctor: That the Senate may understand the present provision I will read a line or two from the act of March 2, 1899, in regard to veterinarians:

> *Provided*, That the veterinarian appointed to the first grade shall not be so appointed until he shall have passed an examination, to be prescribed by the Secretary of War, as to his physical, moral, and professional qualifications.

Now, in regard to this matter, I will say that a bill precisely like the amendment, as I understand it, came before the Military Affairs Committee, and came through the War Department. The Senator from Colorado [Mr. Wolcott] wishes to know what officials there oppose it.

Mr. Wolcott: Yes.

Mr. Proctor: It came through the War Department strongly indorsed, as I recall it (I will have the indorsements in a few minutes), and with the indorsement of the Secretary against the creation of this corps.

I will also say that it seems to me that this bill is not the proper place for this question to be tried. This bill only increases the field artillery, which is the only force that would require veterinarians, by what is the equivalent of a regiment and a half, and four veterinarians are appointed for that force, more than there would be for the same number of cavalry. When there is a general reorganization and increase of the cavalry, of course there will be a large increase; and it seems to me it would be more appropriate to be considered then.

I will say that all the officers of West Point are instructed very carefully in the care and treatment of diseases of the horse, of course.[1]

Mr. Sewell: Mr. President, there has been an effort for two or three years past, last year particularly, on the part of private gentlemen outside to create this corps. I have never known any measure brought before Congress that had so much manufactured support from outside to further it. It proposes to create a colonel, a major, 4 captains, 10 first lieutenants, 20 second lieutenants—36 additions to the veterinary corps of the Army, now consisting of 20. One is to have the rank, pay, and allowance of a second lieutentant and one that of sergeant-major. The Army has existed—

Mr. Lodge: Will the Senator from New Jersey allow me to ask him a question? How many men would these officers whom he has just enumerated have under their command?

[1] Error.—Ed.

Mr. Sewell : There are ten regiments of cavalry.

Mr. Lodge : I mean how many men would be in the corps?

Mr. Sewell : Thirty-six.

Mr. Lodge : They are all officers?

Mr. Sewell : Yes, they are all officers.

Mr. Hale : Just as surgeons are all officers.

Mr. Sewell : I want to say, from long experience in connection with cavalry, that the veterinarian of the cavalry is the captain of the troop. He is the man who knows the condition of his horses. He has been in the cavalry fifteen years—seven or eight years as second lieutentant, seven or eight as first lieutentant—before he becomes a captain. He knows more by reason of his study, and we have the most advanced books on that science; and as his life depends upon his horses and upon having them in good condition, there is no man more interested than the commanding officer of the troop. He is delegated often to buy his own horses by reason of his knowledge; some more, some less.

But he ought not to be interfered with by a lot of youngsters who come out from a college, where they have been taught to dissect, but whose knowledge is not a particle of use to the troop. The old veterinarian, commencing with the farrier, has forgotten more than he would learn of the care of the horse, not in the dissecting of his foot in case it is broken, not in scraping his kidneys or his liver, something that is of no use, because you cannot resurrect a horse after he has broken his leg. He has to go down.

Mr. President, this is just an attempt to legislate thirty-six officers, a new corps, into the Army, without the desire of the Army, without the sanction of the Secretary of War, without any indorsement except the gentlemen who want to fill the positions, very nice ones, to be sure, but without any necessity, in any sense.

They tell us that the armies of Europe all have veterinarians. They have. They have a great many other things we do not want. It would pay the English Government to send over here and get forty or fifty or a hundred farriers to go down there and save their horses now from the veterinarians who graduated from their colleges. We have no use for this. As I say, it is the desire of some gentlemen to have commissions. That is all it is.

Mr. Kenney : In reply to the discussion as to who favors it, or whether there is an indorsement of this amendment by the War Department, I desire to say that from General Miles down, including every head of a bureau in the War Department—I mean the Quartermaster-General, the Inspector-General, General Miles himself, and others whom I cannot now call to mind—they have stood ready and willing to indorse to the Secretary of War, or to the Committee on Military Affairs of the Senate or the House, this proposed legislation if an opportunity had been given to them so to indorse. But on no occasion has the Secretary of War or the Adjutant-General or the Assistant Secretary of War, or, as I understand it, the Committee on Military Affairs of the Senate, called upon General Miles or the Quartermaster-General or the Inspector-General or any of these officers to whom I refer to know whether or not they do or do not indorse this proposed legislation.

On the contrary, I have here introduced as a part of my remarks, letters from General Brooke and other Army officers, too lengthy to read, but which will be printed in the *Record* to-morrow, showing that they do, outside of the indorsement to the Secretary of War or the Committee on Military Affairs, heartily and fully indorse this scheme. That is true all the way down from General Sheridan's time, and had it not been for his untimely death I have not the slightest doubt but that to-day, and long ago, we would have had in the Army of the United States a properly organized and commissioned veterinary corps.

The distinguished Senator from New Jersey [Mr. Sewell] speaks of the interference of these young men of the veterinary corps with the captain of the troop. That cannot be so. He will be the commander of the troop, company, or battery, and these men will have the same relations with him that the surgeons in the army have. There has been no contention that there is friction between the surgeons or assistant surgeons in the Army and the colonels commanding the regiments and the captains of companies or troops or batteries. Then why should there be more friction between these commissioned officers, if they should be made so, than in the case I have referred to in the Medical Department? There will be none.

I desire, in order to show just what great amount of money the United States has invested in Army animals, to read a table from the annual report of the Quartermaster-General of the United States Army in 1899, showing the purchase during the year from July 1, 1898, to June 1, 1899, which was practically after the close of the Spanish war:

| | Number. | Total cost. | Aver. per head. |
|---|---|---|---|
| Cavalry horses . . . | 2094 | $219,727 17 | $104 94 |
| Artillery horses . . . | 775 | 96,374 04 | 124 35 |
| Riding horses . . . | 176 | 22,730 00 | 129 15 |
| Draft horses . . . . | 15 | 2,496 50 | 166 43 |
| Pack horses . . . . | 71 | 3,595 00 | 50 63 |
| Bell horses . . . . | 1 | 50 00 | 50 00 |
| Draft mules . . . . | 3834 | 416,131 50 | 108 54 |
| Pack mules . . . . | 301 | 28,294 00 | 94 00 |
| Oxen . . . . . . | 16 | 1,600 00 | 100 00 |
| Total . . . . | 7283 | $790,998 21 | |

There remained on hand at the close of the fiscal year, June 30, 1899, 12,622 horses and 13,158 mules, valued at over $2,500,000.

In addition to this enormous investment in animals for the cavalry, artillery, and transportation, the Army purchased large quantities of animal food, the quality of which requires inspection.

To show exactly the difference that there will be in the organization of a commissioned corps from that which we have to-day, in cost and number of officers, I desire to submit the following:

For the first year under this amendment, should it become a law, there would be 1 colonel, at $3500 a year; 20 second lieutenants, with salaries amounting to $30,000; 14 quartermaster veterinarians, at salaries amounting to $25,200; making $58,700, as against the present system of $51,000, or a difference after the first year, should the amendment be enacted into law, of $7700.

Now, if you will examine the amendment you will find that there will be a difference after the first year, by promotion from one to the other of the ranks, of more than a thousand dollars. The amendment after the first year would provide for a colonel and 4 captains, 10 first lieutenants, and 20 second lieutenants. That would be a corps consisting of 35, at a cost per annum to the Government of $57,500. The present system after the first year would be 15 civilian quartermaster employés, at $150 a month, or $27,000 per annum; 10 senior veterinarians, at a cost of $15,000 per annum; 10 junior veterinarians, at a cost of $9000 per annum, making $51,000 that the present system will cost the Government against $57,500 after the first year under this organized and commissioned veterinary corps, or a difference of $6500.

Now, can it be successfully contended that there can be, in a comparison of service to the Army, that one is equal to that of the other? It cannot be so. It would be as right to contend that there is no need for a first and second lieutenant in the companies of infantry, the troops of cavalry, and batteries of artillery. They might be non-commissioned officers, if you please. There is no more sense in the one contention than in the other. The gentlemen who go into the Army of the United States and pass the examination required under the provision of this amendment are the sons of gentlemen throughout the country, men who are graduates of the greatest and best universities and colleges in the United States, and they stand shoulder to shoulder as gentlemen and as educated men with any man who comes from West Point or from any other walk of life.

Mr. Proctor: Mr. President, in regard to the reference of this question, I will state that it is not customary for the Committee on Military Affairs to consult all the officers of the Department. There is a regular channel. Bills are referred to the Secretary, and he calls on such officers as have to deal with the subject for their reports. When this bill was before the committee it was referred in the usual course to the War Department, and I will read the indorsement of the Secretary:

War Department, March 26, 1900.

Respectfully returned to the Committee on Military Affairs, United States Senate, calling attention to the foregoing indorsement.

The foregoing indorsement was that of the Adjutant-General, of course, and was largely quotations from the law, showing that the veterinarians were increased in importance by the act of March 2, 1899. The Adjutant-General says:

The act of March 2, 1899, "For increasing the efficiency of the Army of the United States, and for other purposes," provides (Section 2) for two veterinarians in each cavalry regiment, one with the pay and allowances of a second lieutenant of cavalry and one with pay at the rate of $75 per month and the allowances of a sergeant-major, and provides "that the veterinarian appointed to the first grade shall not be so appointed until he shall have passed an examination, to be prescribed by the Secretary of War, as to his physical, moral, and professional qualifications;" and "*Provided further*, That the veterinarians now in service who do not pass such competitive examination shall be eligible to the position of the second class under such rules as are now prescribed by the regulations."

Upon full consideration of the subject at the time it was believed that this provision would provide for efficient veterinarian service for the cavalry regiments.

Now, the Secretary of War says:

The army is urgently in need of more artillery, more staff officers, and more line officers than it now has, and of more soldiers than it will have after the 30th of June, 1901, unless the fighting force of the permanent establishment shall be largely increased by Congress. No bill of this kind ought to pass until these matters of real importance have been provided for. If there is to be any increase, as there should be, it ought to be in the directions indicated. No such rank as is provided for by this bill ought under any circumstances to be given to the veterinary corps. If the country is willing to pay for more colonels and majors and captains, the money should be expended upon the fighting force of the army to furnish opportunity for promotion to the hundreds of gallant fellows who have been enduring hardship and facing death on the battlefield.

Nor do I consider that a separate veterinary corps is desirable. There are already too many separate organizations reporting to the Secretary of War.

That, I believe, everybody will admit as a truism.

One of the evils of our organization is that of multiple command; that in every geographical or technical organization there are so many officers who are not commanded by the commander, but report directly to Washington.

This provides that the chief veterinarian shall report directly to the Secretary of War.

The result is that it is very difficult to fix responsibility as to the condition of anything, and there is a weakened sense of responsibility as to the condition everywhere. The Secretary of War ought not to be responsible for the condition of the horses of a cavalry regiment; the colonel of the regiment should be held responsible for them, and the duties of veterinarian should be performed by men who are under his orders, reporting to him, and not by members of a corps reporting separately to the Secretary of War. The bill is distinctly a step in the wrong direction, and I hope it will not receive favorable consideration. Due provision is made for veterinary service in the bill referred by the Adjutant-General in the first indorsement hereon, to which I beg to invite the attention of the committee.

ELIHU ROOT,
Secretary of War.

Mr. Lodge: Mr. President, it seems to me that there is one very serious objection to this proposition. No one can deny the importance of veterinarians; no one can deny the importance of having well-educated men for those positions; but the objection to which I refer is the tendency to multiply civilian corps and then load them with rank. We have a civilian engineer in the Navy now with the rank of admiral, a man who never went to sea, whose duties are concerned in civil engineering in yards and docks, no doubt a most excellent officer. We have, if I am not mistaken, paymasters who carry so much rank that they cannot go on a ship. There is a tendency to build up these civilian corps, to load them with rank, and then, of course, as has been pointed out in the letter of the Secretary of War, there is just so much less opportunity for the combatant force.

I think, Mr. President, it is a great mistake to go on with this multiplication of civilian corps with this high rank. Here is a whole corps of officers made, beginning with a colonel and coming down to some twenty second lieutenants. I do not think that we want to enter on the increase of the civilian corps attached to the Army and Navy. We ought to give the rank to the men who are in the fighting line, on the ships and on land, and not multiply it for all these various corps. If one comes in, that is a ground for another to come in. I do not believe we should get any better service by this method rather than by giving good veterinarians suitable pay and the recognition to which I freely admit their profession is entirely entitled. Give them a separate rating, if it is so desired, but do not put them in with a colonel at the head responsible only to the Secretary of War, making another conflict of authority in the organization of the Army.

Mr. Gallinger: Mr. President, a single word. When the proposition was presented to the Senate to create a Lieutenant-General for the Army, we were told that it was important to have it done because European nations had lieutenant-generals in their armies. When this proposition is presented to the Senate, we are told by a distinguished member of the Military Committee and a distinguished soldier that we do not want to imitate the methods of European countries and that we could send over some of our farriers to the Transvaal to teach the educated veterinarians of England important lessons in taking care of their horses.

Now, Mr. President, I do not apprehend that that is so. The fact is, as I understand it, that every army in Continental Europe has a corps of veterinarians substantially the same as is proposed in this amendment, and it is clear to my mind that such would not be a fact if they did not find it to their advantage to have these educated men to care for their animals.

The Senator from Colorado [Mr. Wolcott] has covered this question so clearly and so ably that I do not wish to traverse that ground, but I will venture to add that to my mind it is exceedingly important that we should have educated men in our Army to care for the animals. It is a fact, as stated by the Senator from Colorado, that the veterinarians of this country to-day, those who are properly recognized as such, are men of equal culture and equal education to those of the medical profession or the legal profession or of any other profession that is recognized in the United States. They are men who have given a great deal of time to the study of medicine, medicine in its broadest sense, and to all the specialties that are recognized as being necessary to educate a man to understand the diseases of the human family, as well as those of the brute creation.

I know some of these men who are graduates of universities and who have given long years of study in this country and in Europe to qualify them for the performance of their duties as veterinarians. And yet we hear them spoken of, and it has been spoken in my presence to-day in a whisper by more than one Senator, that we propose to give rank and commission to horse doctors. Well, Mr. President, they are horse doctors, I suppose, in the ordinary acceptation of the term, but they are more than that. They are educated men who have an ambition to distinguish themselves in their chosen profession, and that profession is just as honorable as any other.

Mr. Spooner: They are horse doctors in the same sense that other doctors are man doctors.

Mr. Gallinger: Precisely; and they have educated themselves just as thoroughly to discharge their duty as the man doctor has educated himself to discharge his duty.

Now, Mr. President, it seems to me that no valid objection has been raised against this amendment. The Secretary of War, it is true, seems to have reported against it; but I take it that the Secretary of War knows less about this subject than General Sheridan, or General Miles, or the Quartermaster-General, or men who have been educated in military science know about it. Hence I certainly cannot lay as great stress upon his opinion as I lay upon the opinions of those great officers who were thoroughly acquainted with the needs of the Army and who speak from actual observation and experience.

I will esteem it, Mr. President, a privilege to vote for this amendment, and I trust that it shall be adopted.

Mr. Foraker: Mr. President, I do not rise for the purpose of discussing the merit of veterinary surgeons. I think that has been well set forth by those who have spoken in behalf of this amendment. I should like to vote for the amendment if it could be amended so as to avoid creating a separate corps and taking the veterinary surgeons out from under the authority and direction of the regimental and other Army commanders and be so limited as simply to give to them rank. I rise for the purpose of suggesting that to the Senator who has offered this amendment.

I think the men who are to perform this service ought to be recognized as entitled to rank as well as pay. It is proposed by this legislation, as it has already been provided by the legislation that this refers to, to give them the pay and the allowances, and I see no reason why we should not give them the rank as well.

Mr. Kenney: I will suggest, in reply to what the distinguished Senator from Ohio has said, that I will accept an amendment, so that my amendment would read like this:

There shall be in the army the following:

And by striking out in lines 13 and 14:

And the chief veterinarian shall report directly to that officer.

So as to read:

All rules and regulations governing the veterinary corps shall be made by the Secretary of War.

Therefore it would place it entirely in the hands of the Secretary of War to direct how the force should be used and through what channels it should report.

Mr. Spooner: If the Senator will allow me, what provision will he make for rank?

Mr. Kenney: I leave the rank just as it is, but I am willing to strike out the words "That the veterinary corps shall consist of" and substitute in lieu thereof "there shall be attached to the Army a chief veterinarian with the rank, pay, etc.," and then provide for all as my amendment did originally.

Mr. Spooner: What is the rank?

Mr. Kenney: First, a colonel, 4 captains, 10 first lieutenants, and 20 second lieutenants.

Mr. Spooner: That makes a corps. I understood that it was the suggestion of the Senator from Ohio to give these veterinarians now in the service an appropriate rank.

Mr. Kenney: They are provided for, you will find.

Mr. Spooner: Some of us are willing to vote for a proposition like that who are not willing to vote for a colonel, a lieutenant-colonel, a major, and so on.

Mr. Tillman: I ask the Senator from Delaware what use have we for a colonel? If these officers are to be subordinate to the captain or colonel of the cavalry or artillery regiments where they have horses, merely an appendage, so to speak, like any other subordinate officer, what use have we for a colonel anywhere in this business, or any other officer other than the veterinarian who shall rank along with the lieutenant or captain, if we choose, and perform a specific duty? I can see the necessity for some rank, because otherwise the privates will not pay the same respect to orders and will not care for their horses under instructions unless the veterinarian appeals to the officer in command; but I really do not see any use of a corps here, and I do not see any use for any higher officer than lieutenant or possibly captain.

Mr. Foraker: The remarks of the Senator from South Carolina meet the suggestion I made exactly. It is my chief objection to this amendment that it provides a corps and takes a veterinary surgeon who belongs to the regiment out from under the command and authority of the colonel and other officers of that regiment.

Mr. Tillman: It puts somebody supposed to be very skilled in veterinary surgery at the head of a bureau here in Washington to sit down here with a salary and do nothing, and to that I object. I will vote for some rank for these officers and a reasonable number of them, but I do not see any sense in forming a bureau of veterinary surgery here in the War Department.

Mr. Wolcott: I suggest that we vote on the amendment as it stands.

Mr. Kenney: Very well.

The President pro tempore: The Senator from Delaware demanded on this amendment the yeas and nays. Is there a second?

The yeas and nays were ordered.

Mr. Tillman: What is the shape in which the proposition is now to be voted on?

Mr. Kenney: As originally offered.

Mr. Tillman: Just as it was originally offered?

The President pro tempore: The question is on the original amendment without modification. The Secretary will call the roll on agreeing to the amendment of the Senator from Delaware.

The Secretary proceeded to call the roll.

Mr. Burrows (when his name was called): I am paired with the senior Senator from Louisiana [Mr. Caffery].

Mr. Culberson (when Mr. Chilton's name was called): My colleague [Mr. Chilton] is unavoidably absent on account of illness.

Mr. Hansbrough (when his name was called): I again announce my pair with the senior Senator from Virginia [Mr. Daniel], and I will take the liberty of transferring that pair to the senior Senator from Rhode Island [Mr. Aldrich] and will vote. I vote "yea."

Mr. Lindsay (when his name was called): I have a general pair with the senior Senator from Michigan [Mr. McMillan], and withhold my vote.

Mr. Pritchard (when his name was called): I have a general pair with the junior Senator from South Carolina [Mr. McLaurin]. I therefore withhold my vote.

Mr. Proctor (when his named was called): With the consent of the Senator from Utah [Mr. Rawlins], I will make the same transfers of pairs I did before and vote. I vote "nay."

Mr. Spooner (when his name was called): Does the Senator from Tennessee [Mr. Bate] know how his colleague [Mr. Turley] would vote?

Mr. Bate: I do not. He would not object, I am sure, to the Senator's voting on this question.

Mr. Spooner: Then I will vote. I vote "nay."

Mr. Taliaferro (when his name was called): I have a general pair with the junior Senator from West Virginia [Mr. Scott].

Mr. Tillman (when his name was called): I have a general pair with the Senator from Nebraska [Mr. Thurston]. He is absent and I withhold my vote. If I were at liberty to vote, I should vote "nay."

The roll-call was concluded.

Mr. Bacon (after having voted in the affirmative): I have a general pair with the junior Senator from Rhode Island [Mr. Wetmore]. This is a question so purely non-political that I will allow my vote to stand, as I am authorized to do under our agreement.

Mr. Taliaferro: I transfer my pair to the senior Senator from Texas [Mr. Chilton] and vote. I vote "yea."

Mr. Butler: I am paired with the Senator from Maryland [Mr. Wellington], and I therefore withhold my vote.

The result was announced—yeas 25, nays, 23; as follows:

YEAS—25.

| | | | |
|---|---|---|---|
| Allison, | Frye, | McCumber, | Taliaferro, |
| Bacon, | Gallinger, | McEnery, | Teller, |
| Baker, | Gear, | Morgan, | Turner, |
| Chandler, | Hale, | Nelson, | Wolcott. |
| Clay, | Hansbrough, | Perkins, | |
| Culberson, | Kenney, | Quarles, | |
| Foster, | McComas, | Stewart, | |

NAYS—23.

| | | | |
|---|---|---|---|
| Bard, | Foraker, | Pettus, | Shoup, |
| Bate, | Hawley, | Platt, Conn. | Spooner, |
| Cockrell, | Keen, | Proctor, | Sullivan, |
| Deboe, | Kyle, | Rawlins, | Vest, |
| Depew, | Lodge, | Ross, | Warren. |
| Fairbanks, | Pettigrew, | Sewell, | |

NOT VOTING—39.

| | | | |
|---|---|---|---|
| Aldrich, | Clark, Wyo. | Jones, Nev. | Platt, N. Y. |
| Allen, | Cullom, | Lindsay, | Pritchard, |
| Berry, | Daniel, | McBride, | Scott, |
| Beveridge, | Davis, | McLaurin, | Simon, |
| Burrows, | Elkins, | McMillan, | Thurston, |
| Butler, | Hanna, | Mallory, | Tillman, |
| Caffery, | Harris, | Martin, | Turley, |
| Carter, | Heitfeld, | Mason, | Wellington, |
| Chilton, | Hoar, | Money, | Wetmore. |
| Clark, Mont. | Jones, Ark. | Penrose, | |

So Mr. Kenney's amendment was agreed to.

